# Functional Antibodies and Innate Immune Responses to WRSS1, a Live Oral *Shigella sonnei* Vaccine Candidate, in Bangladeshi Adults and Children

**DOI:** 10.1093/infdis/jiab395

**Published:** 2021-08-10

**Authors:** Protim Sarker, Akhirunnesa Mily, Anjuman Ara, Farjana Haque, Nicole Maier, Thomas F Wierzba, Richard I Walker, Malabi M Venkatesan, Rubhana Raqib

**Affiliations:** 1 Infectious Diseases Division, icddr,b, Dhaka,Bangladesh; 2 Center for Vaccine Innovation and Access, PATH, Washington, District of Columbia,USA; 3 Bacterial Diseases Branch, Walter Reed Army Institute of Research, Silver Spring, Maryland,USA

**Keywords:** anti-inflammatory cytokines, host defense peptides, mucosal immune responses, proinflammatory cytokines, protective immunity, serum bactericidal antibody assay

## Abstract

**Background:**

We demonstrated in a randomized placebo-controlled trial that WRSS1, a live oral *Shigella sonnei* vaccine candidate, is safe in Bangladeshi adults and children, and elicits antigen-specific antibodies. Here, we describe functional antibody and innate immune responses to WRSS1.

**Methods:**

Adults (18–39 years) and children (5–9 years) received 3 doses of 3 × 10^5^ or 3 × 10^6^ colony forming units (CFU) of WRSS1 or placebo, 4 weeks apart; children additionally received 3 × 10^4^ CFU. Blood and stool were collected at baseline and 7 days after each dose. Functional antibodies were measured using serum bactericidal antibody (SBA) assay. Cytokine/chemokine concentrations were measured in lymphocyte cultures. Host defense peptides LL-37, HBD-1, and HD-5 were analyzed in plasma and stool.

**Results:**

Children showed increased SBA titers over baseline after the third dose of 3 × 10^6^ CFU (*P* = .048). Significant increases of Th-17 and proinflammatory cytokines (TNF-α, G-CSF, MIP-1β), and reduction of anti-inflammatory and Th2 cytokines (IL-10, IL-13, GM-CSF) were observed in children. Plasma HBD-1 and LL-37 decreased in children after vaccination but were increased/unchanged in adults.

**Conclusions:**

Functional antibodies and Th1/Th17 cytokine responses in children may serve as important indicators of immunogenicity and protective potential of WRSS1.

**Clinical Trials Registration**: NCT01813071.

Development of effective *Shigella* vaccines requires a more comprehensive understanding of the innate and adaptive immune responses that lead to protective immunity. Because *Shigella* is a mucosal pathogen, it is generally believed that protection will require the induction of pathogen-specific B- and T-cell responses and the expression of strong serum and intestinal antibody responses [1–5].

Recent studies exploring potential correlates or markers of anti-*Shigella* protective immunity have focused on antigen specificity and functionality of serum and intestinal antibodies [1, 4, 6, 7]. Most of the current enteric vaccines are known to prevent illness by serum or mucosal antibodies whose functional activity needs to be established. The World Health Organization Expert Committee on Biological Standardization has recommended evaluating the functional antibody responses postvaccination if a proper assay is available [8]. Because antibodies can kill infecting microorganisms through a complement-mediated pathway, serum bactericidal antibody (SBA) assays are often used to evaluate functional antibody responses. For example, serum vibriocidal antibodies constitute the best correlate of protection for oral and parenteral cholera vaccines [9]. An SBA assay was also used to support meningococcal polysaccharide vaccine licensure [10]. Efforts to improve and harmonize methods to measure anti-*Shigella* SBA responses are now in effect [4, 6, 7, 11] and should enable its more widespread application in future *Shigella* vaccine studies. Currently, we have limited data on its application in different age groups receiving a live attenuated vaccine.

*Shigella* is a facultative intracellular organism, and hence cellular immunity is required in addition to antibody-mediated immunity in host defense against this pathogen. In patients with acute *Shigella* infections, differential expression of T helper 1 (Th1) and Th2 cytokines in the rectal mucosa, stool, and plasma correlated with clinical severity of the disease, as well as recovery from illness [12, 13]. It is hypothesized that a distinct T-cell immune signature (eg, cytokines) is generated after natural *Shigella* infection which may be important for protective immunity, and needs to be examined in *Shigella* vaccine trials. Adults immunized with an inactivated whole-cell *Shigella flexneri* 2a vaccine, Sf2aWC, elicited interleukin 17 (IL-17), IL-2, interferon-γ (IFN-γ), tumor necrosis factor-α (TNF-α), and IL-10 in plasma [14]. Live, attenuated *Shigella dysenteriae* type-1 vaccine candidate SC599 also induced enhanced production of IL-17, IL-1β, IL-6, TNF-α, granulocyte colony-stimulating factor (G-CSF), and IFN-γ in healthy adults [15]. Another study showed generation of T-cell–mediated immunity after vaccination with live, attenuated *S. flexneri* 2a vaccine candidate CVD 1208S with T effector memory and central memory subsets being the main cytokine producers [5]. Findings of these studies support the importance of T-cell mediated cytokine responses, which likely complement the humoral responses in adaptive immunity and are worth exploring in future *Shigella* vaccine studies.

Host defense peptides (HDPs), such as defensin and cathelicidin, are important components of the innate immune system [16, 17]. In vaccinated mice, HDPs in the gastric mucosa induced by IL-22 played a key role in protection against *Heliobacter pylori* [18]. Immunization of infants with oral polio vaccine (OPV) within 48 hours of birth resulted in higher enhanced of cathelicidin LL-37 in stools at 6 weeks of age compared to nonimmunized infants [19]. Previous studies have shown that recovery from shigellosis and other diarrhea was linked with restoration of cathelicidin in the intestinal mucosa [20–24].

We have earlier described that WRSS1, a live attenuated *Shigella* vaccine candidate, generates a strong mucosal and systemic immunoglobulin A (IgA) response and more modest immunoglobulin G (IgG) responses, to *Shigella sonnei* lipopolysaccharide in Bangladeshi adults and children [25]. In the present study, the functional characteristics of the antibodies generated by WRSS1 were assessed by performing SBA assays. In addition, cytokines and HDP concentrations were measured to explore further the host innate immunity to WRSS1.

## METHODS

### Study Participants and Study Design

**Figure 1.**
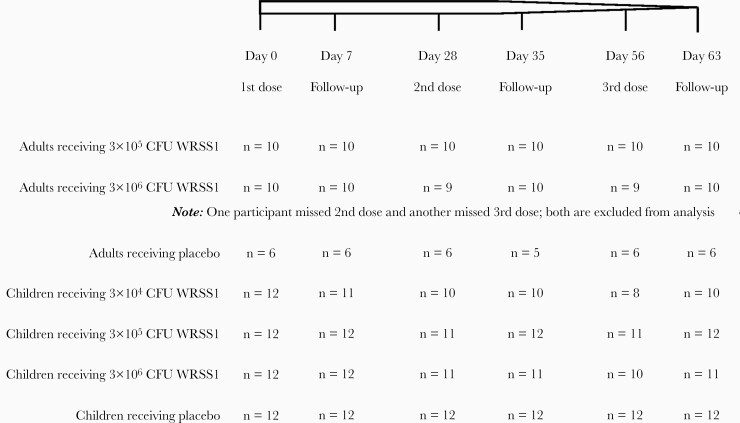
Schematic diagram of dosing and follow-up status of adult and child participants receiving different concentration of WRSS1 vaccine or placebo. Abbreviation: CFU, colony-forming unit.

Specimens were collected from adults (18–39 years) and children (5–9 years), participating in a phase 1 WRSS1 vaccine trial conducted at icddr,b, Dhaka, Bangladesh. Only those participants who received 3 doses of vaccines, 4 weeks apart, and completed all follow-up visits were included for analysis of SBA, cytokines/chemokines, LL-37, human defensin-5 (HD-5), and human β defensin-1 (HBD-1). A brief description of vaccination and follow-up status of adult and child participants are given in Figure 1, and described in detail previously [25]. Ethical clearance was obtained from the ethical review committee of icddr,b and the Western Institutional Review Board for the clinical trial (registered at www.clinicaltrials.gov NCT01813071). All adult participants and guardians of child participants provided written informed consent prior to enrollment.

### Specimen Collection and Processing

Venous blood was collected from participants before immunization and 7 days postvaccination at each dose, that is on day 0, 7, 35, and 63, as previously described [25]. Plasma and serum were separated and stored at –80°C in aliquots. Peripheral blood mononuclear cells (PBMCs) were isolated, incubated in cell culture medium at a concentration of 1 × 10^7^/mL at 37°C, 5% CO_2_ for 72 hours without antigenic stimulation, and the supernatant was collected and stored at −80°C as previously described.

Stool was collected on day 0, 7, 35, and 63 after vaccination at each dose. Extraction of protein-rich fraction from stool has been previously described [25]; the protein-rich filtrate was centrifuged at 12 000*g* for 30 minutes and the supernatant collected and stored at −80°C in aliquots.

### Analysis of Serum Bactericidal Antibody Response

Two to three isolated colonies from a freshly streaked Trypticase soy agar (Becton Dickinson) plate of a *S. sonnei* stock culture were transferred to a culture flask containing  approximately 20 mL tryptic soya broth (Becton Dickinson) enriched with 0.6% yeast extract and grown to exponential phase by incubating at 37°C for 2 hours in a shaker incubator. Bacterial cells were pelleted by centrifugation at 7000 rpm for 10 minutes. After 3 washes, bacterial concentration was adjusted to an optical density (OD) at 595 nm of 0.4 with sterile phosphate-buffered saline, which corresponded to 10^8^ colony forming units (CFU) bacteria/mL. A 10-fold serial dilution was made to prepare a 10^7^ CFU/mL bacterial suspension. Serum samples were incubated at 56°C for 30 minutes to inactivate complement. Two-fold dilutions of serum samples (from 1:8 to 1:512) and guinea pig complement (Sigma-Aldrich) were added to the bacterial suspension in Mueller-Hinton broth (MHB; Becton Dickinson) in wells of microtiter plates in a final volume of 200 µL/well. Control wells contained bacteria in MHB without serum. After overnight incubation (at 37°C for approximately 16 hours at 200 rpm), OD of the bacterial suspension in each well was measured at 595 nm. The SBA titer of the serum was defined as the reciprocal of the last dilution in which no growth was evident by visual inspection.

### Measurement of Cytokines and Chemokines

A 17-plex cytokines and chemokines kit (Bioplex 200 system; Bio-Rad) was used to determine the concentrations (pg/mL) of cytokines and chemokines in the lymphocyte supernatant following manufacturer’s instructions. The kit contained: IL-1β, IL-2, IL-4, IL-5, IL-6, IL-7, IL-8, IL-10, IL-12(p70), IL-13, IL-17, G-CSF, granulocyte-macrophage colony-stimulating factor (GM-CSF), IFN-γ, monocyte chemoattractant protein-1 (MCP-1), macrophage-inflammatory protein-1β (MIP-1β), and TNF-α.

### Measurement of Host Defense Peptides

Levels of HDPs LL-37 (ng/mL), HBD-1 (pg/mL), and HD-5 (pg/mL) were measured in plasma and stool extracts by using enzyme-linked immunosorbent assay (ELISA) kits (LL37, Hycult Biotech; HBD-1, Alpha Diagnostic International; HD-5, Elabscience) according to manufacturers’ instructions.

### Statistical Analysis

Statistical analysis was performed with Stata/IC version 13 (Stata Corp). Figures were prepared using GraphPad Prism 7.0. Data are presented as median and interquartile range. Statistical significance between pre- and postvaccination days within group was determined using Wilcoxon signed rank test. Kruskal-Wallis test was applied to compare vaccine groups with placebo at each time point. Statistical comparison of baseline variables between adults and children was determined using Mann-Whitney *U* test for unequal samples. A *P* value < .05 was considered significant.

## RESULTS

### Baseline Comparison of SBA Titers, Cytokines/Chemokines, and HDPs in Adults and Children

The baseline titers/levels of SBA, cytokines/chemokines, and HDPs differed between adult and child participants. SBA titers and levels of IL-17, TNF-α, MIP-1β, G-CSF, GM-CSF, LL-37, and HBD-1 were higher in adults, while IL-7 concentration was higher in children (Table 1).

**Table 1.** Comparison of Baseline SBA Titers, and Levels of Cytokines and Host Defense Peptides Between Adults and Children

Variables	Adults	Children	P Value
SBA	57.7 (55.3–118.1)	32 (16–32)	.003
IL-7	11.1 (9.2–12.4)	23.2 (16.3–35.2)	<.001
IL-17	155.6 (104–240)	111 (87.4–143.8)	.003
TNF-α	509.4 (311–997)	212.4 (157–286)	<.001
MIP-1β	222 (105–663)	65.8 (29.0–167)	<.001
G-CSF	2494 (1344–7656)	590 (334–1908)	.001
IL-10	28.2 (23.1–42.2)	29.4 (16.9–42.9)	.437
IL-13	5.82 (4.68–9.1)	4.7 (3.0–7.0)	.053
GM-CSF	355 (295–381)	54.7 (20.0–102)	<.001
LL-37	3.08 (2.50–3.56)	2.38 (1.81–2.95)	.003
HBD-1	509 (467–631)	337 (297–395)	<.001

Data are median (interquartile range). Statistical significance was determined using Mann-Whitney *U* test for unequal samples.

Abbreviations: G-CSF, granulocyte colony-stimulating factor; GM-CSF, granulocyte-macrophage colony-stimulating factor; HBD-1, human β defensin-1; IL, interleukin; MIP-1β, macrophage inflammatory protein-1β; SBA, serum bactericidal antibody; TNF-α, tumor necrosis factor-α.

### SBA Responses in Adults and Children After Vaccination With WRSS1

**Figure 2.**
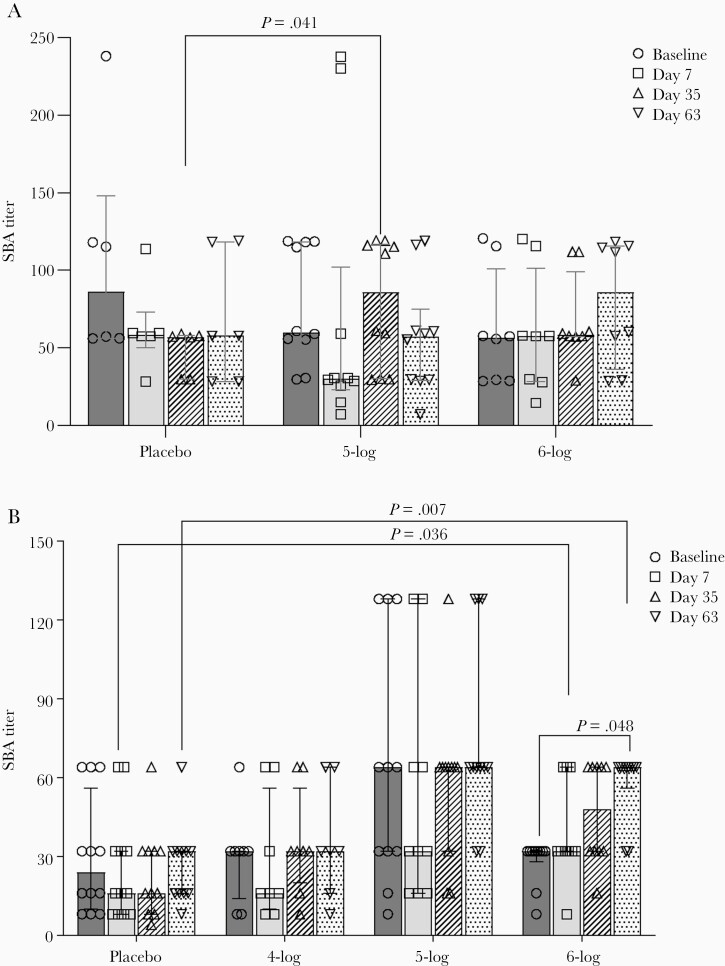
Serum bactericidal antibody (SBA) titers before and after administration of WRSS1vaccine or placebo in Bangladeshi adults (*A*) and children (*B*). Data are presented as median and interquartile range. Statistical significance between pre- and postvaccination days within group was determined using Wilcoxon Signed rank test. Kruskal-Wallis test was applied to compare vaccine groups with placebo at each time point.

Among adults, there was no change in SBA titers over baseline after any dose of WRSS1 vaccination. However, titers in the 5-log group were significantly higher than in the placebo group at day 7 after the second vaccination (day 35, *P* = .041; Figure 2A). When SBA titers were compared between shedders and nonshedders, WRSS1 shedders in the highest dose (6-log) group exhibited higher titers than nonshedders (*P* = .049) after the third dose (day 63) (data not shown). In children, the SBA titers increased significantly in the 6-log group 7 days after the third dose of vaccination (day 63) compared to baseline (*P* = .048). Compared to placebo, SBA titers in the 6-log group were significantly higher 7 days after the first and third vaccination (day 7, *P* = .036; day 63, *P* = .007; Figure 2B).

### WRSS1 Vaccination in Adults Induced IL-7

**Figure 3.**
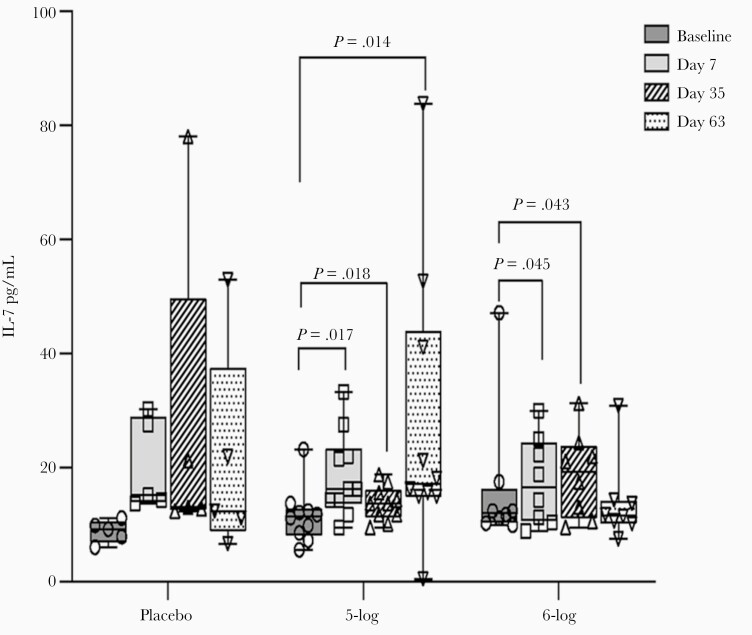
Interleukin 7 (IL-7) concentration in lymphocyte culture of Bangladeshi adult participants before and after administration of WRSS1 vaccine or placebo. Data are presented as median and interquartile range. Statistical significance between pre- and postvaccination days within group was determined using Wilcoxon Signed rank test. Kruskal-Wallis test was applied to compare vaccine groups with placebo at each time point.

IL-7 levels in adults showed an increase over baseline after every dose in the 5-log group. In the 6-log group, the increase was statistically significant after the first and second doses (day 7, *P* = .045; day 35, *P* = .043; Figure 3). However, when compared with placebo, the increase in the IL-7 levels in vaccinees was not significant on any day. Vaccination did not affect any other cytokines tested in adults.

### WRSS1 Vaccination in Children Augmented Proinflammatory and Downregulated Anti-inflammatory and Th2 Cytokines

Vaccination in children induced a significant rise in IL-17 levels over baseline after the second vaccination (day 35) in the 5-log group (*P* = .046) and after the third vaccination (day 63) in the 6-log group (*P* = .014). In the 4-log group, although IL-17 increased gradually after vaccination, the difference over baseline was not significant (Table 2). When compared to placebo, IL-17 levels were significantly higher at day 35 (*P* = .03) in the 5-log group; the levels were also higher in the 6-log group at day 63, although the difference was not statistically significant.

**Table 2.** Concentrations of Th17 and Proinflammatory Cytokines in Culture Supernatant in Children Before and After Vaccination With WRSS1

		Concentration, pg/mL			
Cytokine	Dose Group	Baseline	Day 7	Day 35	Day 63
IL-17	Placebo (n = 12)	108.3 (98.1–129.6)	133.7 (83.3–147.5)	118.8 (86.1–144.8)	132.9 (93.6–209.4)
	4-log (n = 8)	109.4 (79.2–156.2)	117.2 (84.8–154.1)	129.1 (116.4–153.2)	166.1 (157.9–186.3)
	5-log (n = 11)	72.9 (34.5–143.5)	99.5 (68.7–147.3)	159.5 (125.4–207.4)*^§^	107.9 (54.7–127.5)
	6-log (n = 10)	115.5 (100.9–146.5)	97.3 (86.0–118.8)	87.9 (70.4–118.8)	221.1 (141.7–285.7)*
TNF-α	Placebo (n = 12)	211.3 (190.9–276.3)	223.5 (153.1–303.4)	200.0 (162.6–306.8)	235.9 (190.4–406.6)
	4-log (n = 8)	218.2 (166.7–358.0)	254.1 (189.1–378.1)	256.9 (237.8–287.4)	373.8 (304.2–472.3)
	5-log (n = 11)	139.0 (57.7–296.0)	173.3 (84.7–252.7)*	335.8 (288.9–372.0)*^§^	214.6 (121.4–288.1)
	6-log (n = 10)	226.2 (169.3–265.4)	175.9 (150.2–211.8)	158.7 (117.7–238.4)	325.5 (216.8–805.8)*
MIP-1β	Placebo (n = 12)	84.3 (50.4–228.1)	69.3 (55.7–228.6)	158.6 (65.8–294.0)	207.4 (26.7–613.8)
	4-log (n = 8)	45.7 (27.2–57.4)	161.1 (24.6–328.7)	112.9 (68.1–122.8)	219.9 (63.7–460.0)*
	5-log (n = 11)	25.3 (17.3–63.8)^§^	72.6 (55.7–401.6)*	162.4 (24.9–208.9)*	31.5 (9.2–179.5)
	6-log (n = 10)	159.8 (94.3–261.0)	227.4 (154.9–307.5)*^§^	234.3 (142.2–268.2)	490.8 (201.8–662.1)*
G-CSF	Placebo (n = 12)	775 (416–2227)	675 (385–2218)	595 (316–2375)	1319 (642–3542)
	4-log (n = 8)	1830 (1208–3106)	1998 (983–2747)^§^	2171 (1785–2866)	4853 (2757–7276)*^§§§^
	5-log (n = 11)	561 (98–1978)	1070 (172–1740)	2190 (1503–3294)*	824 (308–2066)
	6-log (n = 10)	484 (377–596)	471 (338–579)	324 (141–535)	1060 (330–1499)*

Data are median (interquartile range). Statistical significance between pre- and postvaccination days within group was determined using Wilcoxon Signed rank test. Kruskal-Wallis test was applied to compare vaccine groups with placebo at each time point. *P ≤ .05 when comparison was made between pre- and postvaccination days. ^§^*P* ≤ .05; ^§§§^*P* ≤ .005 when vaccine groups were compared with placebo.

Abbreviations: G-CSF, granulocyte colony-stimulating factor; IL-17, interleukin 17; MIP-1β, macrophage inflammatory protein-1β; TNF-α, tumor necrosis factor-α.

The levels of TNF-α and MIP-1β showed a significant increase over baseline in the 5-log group after the first (day 7, *P* < .03) and second (day 35, *P* < .035) dose, respectively. In the 6-log group, a significant increase was only seen for MIP-1β after the first dose (day 7, *P* = .05) and for both cytokines after the third dose (day 63, *P* = .03). Compared to placebo, TNF-α levels in the 5-log group were higher at day 35 (*P* = .037) and MIP-1β levels were elevated in the 6-log group at day 7 (*P* = .02). Although TNF-α or MIP-1β levels also increased after vaccination in the 4-log group, the difference over baseline was not significant. G-CSF concentrations increased significantly over baseline at day 63 in the 4-log (*P* = .038) and the 6-log (*P* = .041) groups and at day 35 in the 5-log group (*P* = .039) (Table 2). Compared to placebo, G-CSF levels were higher at day 7 (*P* = .035) and day 63 (*P* = .003) in the 4-log group (Table 2).

Levels of anti-inflammatory cytokine IL-10 declined in the 6-log group after the first vaccination (day 7, *P* = .039) and continued after the second vaccination (day 35, *P* = .001) (Table 3). A significant decline was also seen for IL-13 levels after the third vaccination (day 63, *P* = .004) in the 4-log group and after the second (day 35, *P* < .001) and third vaccination (day 63, *P* < .001) in the 6-log group. WRSS1 vaccination in children led to a profound reduction in GM-CSF levels over baseline after the first (day 7, *P* = .029) and second (day 35, *P* = .002) vaccination in the 6-log group (Table 3). Compared to placebo, the levels of GM-CSF in the 6-log group remained lower (*P* < .05) after all 3 doses (Table 3). No significant changes in cytokine levels were seen in placebo recipients (Table 2 and Table 3). The levels of other cytokines tested in children remained unchanged.

**Table 3.** Concentrations of Anti-inflammatory and Th2 Cytokine in Culture Supernatant in Children Before and After Vaccination With WRSS1

		Concentration, pg/mL			
Cytokines	Dose Group	Baseline	Day 7	Day 35	Day 63
IL-10	Placebo (n = 12)	25.4 (16.9–37.8)	20.9 (9.0–31.6)	11.8 (5.8–24.3)	28.3 (13.2–48.3)
	4-log (n = 8)	46.3 (37.2–55.8)^§§^	29.4 (22.6–34.0)*	47.2 (32.6–82.0)^§§^	50.4 (41.9–64.6)*^§§^
	5-log (n = 11)	17.6 (6.7–32.0)	20.6 (9.4–30.1)	28.3 (19.0–44.4)	18.0 (11.2–31.8)
	6-log (n = 10)	38.9 (14.5–44.7)	15.9 (14.0–20.1)*	11.1 (3.1–15.8)****	42.3 (15.5–73.0)
IL-13	Placebo (n = 12)	6.1 (3.3–7.0)	3.3 (2.1–6.9)	2.7 (2.1–4.3)	3.0 (1.2–5.0)
	4-log (n = 8)	8.7 (5.7–11.6)	6.3 (5.5–6.7)	5.5 (5.0–5.8)^§§§^	4.3 (3.3–5.2)***
	5-log (n = 11)	3.5 (2.7–4.7)^§^	2.8 (2.1–5.2)	4.3 (3.2–5.2)	5.4 (3.3–6.6)^§^
	6-log (n = 10)	3.8 (2.4–5.5)	2.4 (1.8–2.7)	1.7 (1.2–2.5)****^§^	2.3 (0.8–2.9)****
GM-CSF	Placebo (n = 12)	70.9 (9.6–97.9)	74.9 (14.7–118.9)	61.2 (2.5–99.1)	76.7 (33.3–112.9)
	4-log (n = 8)	102.5 (78.5–118.0)^§^	97.3 (74.4–123.6)	92.3 (88.9–108.6)	116.4 (95.9–137.7)^§§^
	5-log (n = 11)	82.1 (13.5–101.4)	73.0 (23.8–95.6)	101.4 (65.0–123.6)	53.2 (33.9–97.3)
	6-log (n = 10)	25.6 (17.1–32.0)	5.8 (2.5–8.7)*^§§^	10.4 (2.5–22.2)***^§^	40.5 (7.4–46.3)^§^

Data are median (interquartile range). Statistical significance between pre- and postvaccination days within group was determined using Wilcoxon Signed rank test. Kruskal-Wallis test was applied to compare vaccine groups with placebo at each time point. *P ≤ .05; ***P ≤ .005; ****P ≤ .001 when comparison was made between pre- and postvaccination days. ^§^*P* ≤ .05; ^§§^*P* ≤ .01; ^§§§^*P* ≤ .005 when vaccine groups were compared with placebo.

Abbreviations: GM-CSF, granulocyte-macrophage colony-stimulating factor; IL, interleukin.

### Differential Effects of Vaccination on Plasma Level of HDPs in Adults and Children

LL-37 and HBD-1 were detected in plasma but not in fecal samples from both adults and children. In contrast, HD-5 could only be detected in fecal samples.

**Figure 4.**
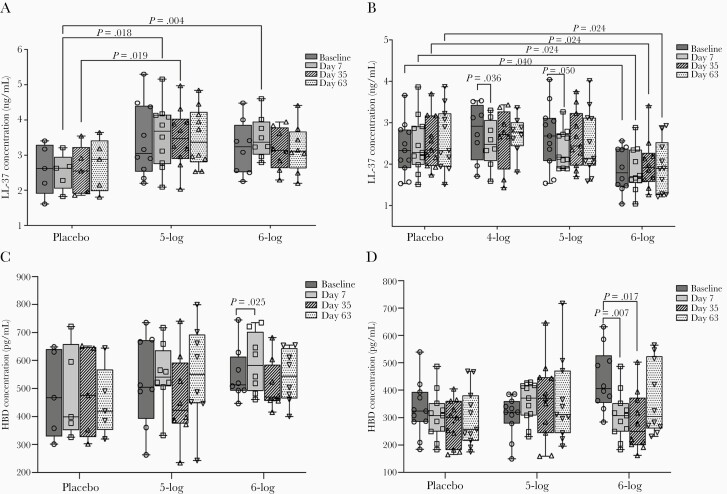
Plasma levels of LL-37 in adults (*A*), LL-37 in children (*B*), human β defensin-1 (HBD-1) in adults (*C*), and HBD-1 in children (*D*) before and after administration of WRSS1 vaccine or placebo. Data are presented as median and interquartile range. Statistical significance between pre- and postvaccination days within group was determined using Wilcoxon Signed rank test. Kruskal-Wallis test was applied to compare vaccine groups with placebo at each time point.

In adults, there was no change in plasma LL-37 levels over baseline at any time in the 5-log and 6-log groups. However, when compared to placebo, plasma LL-37 levels were higher after the first dose (day 7) in the 5-log (*P* = .018) and 6-log groups (*P* = .004) and after the second dose (day 35) in the 5-log group (*P* = .019) (Figure 4A). In children, plasma LL-37 concentration declined significantly in the 4-log (*P* = .036) and 5-log (*P* = .05) groups at day 7 after the first dose (Figure 4B). Significantly lower levels of plasma LL-37 compared to placebo were also observed in the 6-log group throughout the study period (Figure 4B).

HBD1 levels in adults increased significantly over baseline in the 6-log group after the first dose (day 7, *P* = .025) (Figure 4C), while in children the levels declined significantly in the 6-log group after the first (day 7, *P* = .007) and second dose (day 35, *P* = .017) (Figure 4D). No changes were seen in the stool concentrations of HD-5 in either adults or children after vaccination (data not shown).

## Discussion

In endemic countries like Bangladesh, infants and children younger than 5 years of age are the target population for a *Shigella* vaccine. Adults are considered generally immune, a feature attributed to repeated exposure to these pathogens and immunity in children 5–9 years of age may reflect a situation in between adults and infants. In this study, vaccination with WRSS1 induced SBA in 5 to 9-year-old children but not in adults. In the same group of children, Th-17 and proinflammatory cytokines were increased, while anti-inflammatory and Th2 cytokines were diminished postvaccination. IL-7 levels were only increased in adults. Host defense peptide HBD-1 in plasma increased in adults after vaccination, while in children plasma levels of LL-37 and HBD-1 decreased during vaccination.

We have previously demonstrated the antibacterial activity of convalescent serum from *Shigella*-infected adult patients [26]. *S. flexneri*-infected children also showed SBA response against *S. flexneri*, and zinc supplementation resulted in a significant increase in this activity [27]. In a controlled human infection model study with a virulent *S. sonnei* strain 53G, SBA levels increased over baseline by day 7 and peaked at 14 days postchallenge [6]. The SBA titers remained elevated up to day 56 regardless of shigellosis outcome. Elevated levels of baseline SBA correlated with better outcomes after 53G challenge. Immunization of Israeli and North American adults with the conjugate vaccine candidates SF2a-TT15 and Flexyn2a also resulted in robust SBA titers [28, 29]. In a subsequent challenge study with Flexyn2a, higher SBA titers prechallenge correlated with protection from shigellosis [7], suggesting that SBA titers could be an immune marker for protection. Protection against many infectious diseases was proposed to be conferred by induction of high levels of serum IgG antibodies that exude onto mucosal surfaces and kill or inactivate the pathogens [30]. In the present study, children vaccinated with WRSS1 showed an increase in existing SBA titers, while no change in the titers were seen in adults after vaccination. The lack of response in Bangladeshi adults might be due to high preexisting SBA titers (Table 1), probably attained through longer preexposure to *Shigella*. Earlier, we showed that vaccination of children with WRSS1 resulted in a modest increase of antigen-specific serum IgG titers [25]. Together these findings suggest that oral vaccination in children has the potential to impact antibody-mediated functional immunity.

Vaccination of US adults with live attenuated *Shigella* vaccines, including *S. flexneri* 2a (CVD 1207), *S. dysenteriae* 1 (SC595), and WRSS1, elicited type 1 cytokine response in the culture supernatant of PBMCs after stimulation with *Shigella* antigens [31–33]. In a phase 2 clinical trial, adult volunteers receiving live attenuated *S. dysenteriae* type 1 vaccine candidate SC599 showed augmented levels of Th17 cytokine (IL-17) in addition to Th1 cytokines (IL-1β, IL-6, TNF-α, G-CSF, and IFN-γ) in stimulated PBMCs [15]. Upregulation of IL-17, IFN-γ, and TNF-α was also shown in stimulated PBMCs obtained from the recipients of a live attenuated *S. flexneri* 2a vaccine candidate (CVD 1208S), and CD4 and CD8 effector memory T cells were identified as the main subsets producing these cytokines [5]. In addition, serum levels of IL-17, IFN-γ, and TNF-α were increased in nonendemic adults after oral immunization with inactivated *S. flexneri* 2a vaccine candidate (Sf2aWC) [14]. In the present study, WRSS1 vaccination led to release of proinflammatory cytokines (TNF-α, MIP-1β, and G-CSF) and IL-17 in culture supernatant of PBMCs in children. Adult volunteers did not show Th1 and Th17 responses to WRSS1, probably because of high baseline concentrations in the lymphocyte supernatants, as compared to children who had significantly lower baseline concentrations of these cytokines (Table 1). In line with this, we previously showed that pediatric patients with acute shigellosis experience delayed and reduced humoral immune responses, including proinflammatory cytokines, compared to their adult counterpart [34]. Similarly, innate immune cells and their mediators demonstrated delayed accumulation in the rectal tissue of child patients, which persisted for prolonged periods [35]. Here, proinflammatory cytokines, although induced in children, never quite reached adult levels, probably due to the relatively immature nature of the immune system in children.

Elevated levels of TNF-α seen here in children after WRSS1 vaccination is supported by a previous study, where experimental challenge with *S. sonnei* induced TNF-α in naive US volunteers [36]. The role of TNF-α in limiting *Shigella* infection is still unclear; however, this cytokine was shown to reduce luminal content as well as systemic dissemination of *Listeria monocytogenes* in infected mice [37]. The function of chemokine MIP-1β and growth factor G-CSF has also not been established in protective immune response to *Shigella*. IL-17A production from primed Th17 cells after reinfection with *S. flexneri* had a significant role in bacterial clearance in a murine pulmonary model [38]. We earlier showed that IL-10 levels were not detectable in serum or stool of patients during acute and convalescent phases of shigellosis [13]; however, expression of the cytokine in rectal tissue was prominent during both phases [12]. Vaccination of adult volunteers with live attenuated *S. flexneri* 2a and *S. dysenteriae* vaccine candidates induced IL-10 levels in culture supernatants of *Shigella* antigens-stimulated PBMC [14, 15, 31, 33]; however, no change was observed after vaccination with WRSS1. In the present study, the decrease in Th2 cytokines (IL-10 and IL-13) within a proinflammatory cytokine environment (TNF-α and MIP-1β) following WRSS1 vaccination in children is consistent with the profile needed to prime Th17 cells [39]. Whether a similar profile is required for generating protective immunity in children needs to be examined in future *Shigella* vaccine trials.

IL-7 increased after WRSS1 vaccination in adults but not in children. IL-7 is involved in the survival and activation of T and B cells [40, 41]. Activation of T and B cells and increase in T-memory cells was reported previously during natural *Shigella* infection [42]. The role of IL-7 in maintenance and activation of T and B cells after natural *Shigella* infection as well as after vaccination needs to be evaluated in future studies.

Previous studies have shown that neonates receiving OPV and BCG vaccines have higher excretion of LL-37 in feces after vaccination [19]. On the other hand, oral administration of typhoid and Enterotoxigenic Escherichia coli (ETEC) vaccine suppressed constitutive expression of LL-37, HD-5, and HD-6 transcripts in small intestinal biopsies, but had no effect on HBD-1 expression in adult volunteers [43]. In the present study, LL-37 and HBD-1 could only be detected in the plasma, and vaccination with WRSS1 in children downregulated the plasma concentrations of LL-37 and HBD-1. In a landmark study, Islam et al showed that constitutive expression of LL-37 and HBD-1 is downregulated in intestinal epithelial cells of patients during acute shigellosis [44]. This downregulation may be related to facilitation of bacterial spread within the host. In in vitro and xenograph models, the *Shigella* MxiE transcriptional regulator that regulates the host cytosolic expression of genes coding for several type III secreted proteins, such as *ospB*, *ospC1*, *ospE2*, *ospF*, *virA*, and *ipaH*, has been identified as the mediator for the suppression of HDPs by *S. flexneri* [45]. A similar mechanism may exist to explain the downregulation of LL-37 and HBD-1 in children following WRSS1 (which has a VirG deletion) vaccination. In primed adults with existing trained immunity [46], these factors may play a limited role.

One major limitation of the study is the small sample size. Specimens were collected from participants enrolled in a phase 1 clinical trial and only those participants who received all 3 doses and completed all follow-up visits were included in analysis. Another limitation is the variability of the cytokine data between days, which although this did not show significant changes in placebo recipients might indicate nonspecific induction/suppression of the cytokines.

In conclusion, vaccination with WRSS1 induced functional antibody responses in Bangladeshi children. Vaccination in children also induced Th1/Th17 signature cytokines. Vaccination studies in larger populations of young children and infants living in *Shigella-*endemic areas are needed to substantiate the findings reported here and to establish the contribution of these factors to protection against *Shigella*.

## Notes

***Acknowledgments.*** We acknowledge the study participants for their consent to participate in the clinical trial and providing blood and stool specimens. We express our sincere thanks to Mr Ahsanul Haque for his assistance in data analysis for the manuscript.

***Author contributions.*** P. S., M. V., and R. R. conceived and designed the study. P. S., A. M., A. A., and F. H. performed the experiments. P. S., N. M., T. F. W., R. I. W., M. V., and R. R. interpret the results. P. S. and R. R. analyze the data and wrote the manuscript. All authors contributed to the critical review and revision of the manuscript and have approved the final version.

***Disclaimer.*** The opinions or assertions contained herein are the private views of the authors and are not to be construed as official or as reflecting true views of the US Department of the Army or the Department of Defense. The investigators have adhered to the policies for protection of human subjects as prescribed in AR 70-25. PATH participated in the design of the studies, interpretation of results and review of this manuscript.

***Financial support.*** This work was supported by PATH, an international nonprofit organization that works to improve the health of people around the world by advancing technologies, strengthening systems, and encouraging healthy behaviors. Core/unrestricted support for icddr,b was provided by the Government of Bangladesh, Government of Canada, Government of Sweden, and Government of the UK.

***Potential conflicts of interest.*** All authors: no reported conflicts of interest. All authors have submitted the ICMJE Form for Disclosure of Potential Conflicts of Interest. Conflicts that the editors consider relevant to the content of the manuscript have been disclosed.

Presented in part: Second International Vaccines Against *Shigella* and Enterotoxigenic *Escherichia coli* (VASE) Conference, Mexico City, Mexico, 12–14 June 2018.
